# Patient and Staff Experience of Remote Patient Monitoring—What to Measure and How: Systematic Review

**DOI:** 10.2196/48463

**Published:** 2024-04-22

**Authors:** Valeria Pannunzio, Hosana Cristina Morales Ornelas, Pema Gurung, Robert van Kooten, Dirk Snelders, Hendrikus van Os, Michel Wouters, Rob Tollenaar, Douwe Atsma, Maaike Kleinsmann

**Affiliations:** 1 Department of Design, Organisation and Strategy Faculty of Industrial Design Engineering Delft University of Technology Delft Netherlands; 2 Department of Sustainable Design Engineering Faculty of Industrial Design Engineering Delft University of Technology Delft Netherlands; 3 Walaeus Library Leiden University Medical Center Leiden Netherlands; 4 Department of Surgery Leiden University Medical Center Leiden Netherlands; 5 National eHealth Living Lab Department of Public Health & Primary Care Leiden University Medical Center Leiden Netherlands; 6 Department of Surgery Netherlands Cancer Institute – Antoni van Leeuwenhoek Amsterdam Netherlands; 7 Department of Cardiology Leiden University Medical Center Leiden Netherlands

**Keywords:** remote patient monitoring, telemonitoring, patient experience, staff experience, monitoring, intervention, health outcome, adherence, patient monitoring

## Abstract

**Background:**

Patient and staff experience is a vital factor to consider in the evaluation of remote patient monitoring (RPM) interventions. However, no comprehensive overview of available RPM patient and staff experience–measuring methods and tools exists.

**Objective:**

This review aimed at obtaining a comprehensive set of experience constructs and corresponding measuring instruments used in contemporary RPM research and at proposing an initial set of guidelines for improving methodological standardization in this domain.

**Methods:**

Full-text papers reporting on instances of patient or staff experience measuring in RPM interventions, written in English, and published after January 1, 2011, were considered for eligibility. By “RPM interventions,” we referred to interventions including sensor-based patient monitoring used for clinical decision-making; papers reporting on other kinds of interventions were therefore excluded. Papers describing primary care interventions, involving participants under 18 years of age, or focusing on attitudes or technologies rather than specific interventions were also excluded. We searched 2 electronic databases, Medline (PubMed) and EMBASE, on February 12, 2021.We explored and structured the obtained corpus of data through correspondence analysis, a multivariate statistical technique.

**Results:**

In total, 158 papers were included, covering RPM interventions in a variety of domains. From these studies, we reported 546 experience-measuring instances in RPM, covering the use of 160 unique experience-measuring instruments to measure 120 unique experience constructs. We found that the research landscape has seen a sizeable growth in the past decade, that it is affected by a relative lack of focus on the experience of staff, and that the overall corpus of collected experience measures can be organized in 4 main categories (service system related, care related, usage and adherence related, and health outcome related). In the light of the collected findings, we provided a set of 6 actionable recommendations to RPM patient and staff experience evaluators, in terms of both what to measure and how to measure it. Overall, we suggested that RPM researchers and practitioners include experience measuring as part of integrated, interdisciplinary data strategies for continuous RPM evaluation.

**Conclusions:**

At present, there is a lack of consensus and standardization in the methods used to measure patient and staff experience in RPM, leading to a critical knowledge gap in our understanding of the impact of RPM interventions. This review offers targeted support for RPM experience evaluators by providing a structured, comprehensive overview of contemporary patient and staff experience measures and a set of practical guidelines for improving research quality and standardization in this domain.

## Introduction

### Background and Aim

This is a scenario from the daily life of a patient:

A beeping sound, and a message appears on the smartphone screen: “Reminder: check glucose before bedtime.” Time to go to sleep, indeed, you think while putting down your book and reaching for the glucometer. As you wipe the drop of blood away, you make sure that both Bluetooth and Wi-Fi are on in your phone. Then, the reading is sent: you notice it seems to be rather far from your baseline. While you think of what you might have done differently, a slight agitation emerges: Is this why you feel so tired? The phone beeps again: “Your last glucose reading seems atypical. Could you please try again? Remember to follow these steps.” Groaning, you unwrap another alcohol wipe, rub your finger with it, and test again: this time, the results are normal.

Some patients will recognize certain aspects of this scenario, particularly the ones using a form of remote patient monitoring (RPM), sometimes referred to as remote patient management. RPM is a subset of digital health interventions that aim to improve patient care through digitally transmitted, health-related patient data [1]. Typically, RPM interventions include the use of 1 or more sensors (including monitoring devices, wearables, or implants), which collect patient data in or out of the hospital to be used for remote clinical decision-making. Partly due to a rapid expansion during the COVID-19 pandemic [2-5], the RPM domain has by now expanded to reach a broad range of medical specialties, sensing technologies, and clinical contexts [1,6,7].

RPM is presented as a strategy for enabling health care systems worldwide to face the pressing challenges posed by aging populations [8-10], including the dwindling availability of health care workers [11] and rising health care costs [12]. This is because deploying effective RPM solutions across health systems holds the potential to reduce health care resources use, while maintaining or improving care quality. However, evidence regarding RPM effectiveness at scale is mixed [13]. Few large-scale trials demonstrating a meaningful clinical impact of RPM have been conducted so far, and more research is urgently needed to clarify and address determinants of RPM effectiveness [7].

Among these determinants, we find the experience of patients and staff using RPM interventions. As noticeable in the introductory scenario, RPM introduces radical experiential changes compared to in-person care; patients might be asked to download and install software; pair, charge, and wear monitoring devices; submit personal data; or attend alerts or calls, all in the midst of everyday life contexts and activities. Similarly, clinical and especially nursing staff might be asked to carry out data analysis and administrative work and maintain remote contact with patients, often without a clear definition of roles and responsibilities and in addition to usual tasks [14].

Because of these changes, patient and staff experience constitutes a crucial aspect to consider when evaluating RPM interventions. Next to qualitative methods of experience evaluation, mixed and quantitative methods are fundamental, especially to capture information from large pools of users. However, the current RPM experience-measuring landscape suffers from a lack of methodological standardization, reflected in both what is measured and how it is measured. Regarding what is measured, it has been observed that a large number of constructs are used in the literature, often without a clear specification of their significance. This can be noticed even regarding popular constructs, such as satisfaction: Mair and Whitten [15], for instance, observe how the meaning of the satisfaction construct is seldom defined in patient surveys, leaving readers “unable to discern whether the participants said they were satisfied because telemedicine didn't kill them, or that it was ‘OK,’ or that it was a wonderful experience.” Previous work also registers a broad diversity in the instruments used to measure a specific construct. For instance, in their review of RPM interventions for heart failure, Kraai et al [16] report that none of the papers they examined used the same survey to measure patient satisfaction, and only 1 was assessed on validity and reliability.

In this proliferation of constructs and instruments, no comprehensive overview exists of their application to measuring patient and staff experience in the RPM domain. The lack of such an overview negatively affects research in this domain in at least 2 ways. At the level of primary research, RPM practitioners and researchers have little guidance on how to include experience measuring in their study designs. At the level of secondary research, the lack of consistently used measures makes it hard to compare results between different studies and RPM solutions. Altogether, the lack of standardization in experience measuring constitutes a research gap that needs to be bridged in order for RPM to fully deliver on its promises.

In this review, this gap is addressed through an effort to provide a structured overview of patient and staff experience constructs and instruments used in RPM evaluation. First, we position the role of RPM-related patient and staff experience within the broader system of care using the Quadruple Aim framework. Next, we describe the systematic review we performed of patient and staff experience–relevant constructs and instruments used in contemporary research aimed at evaluating RPM interventions. After presenting and discussing the results of this review, we propose a set of guidelines for RPM experience evaluators and indicate directions for further research.

### The Role of Patient and Staff Experience in RPM

**Figure 1:**
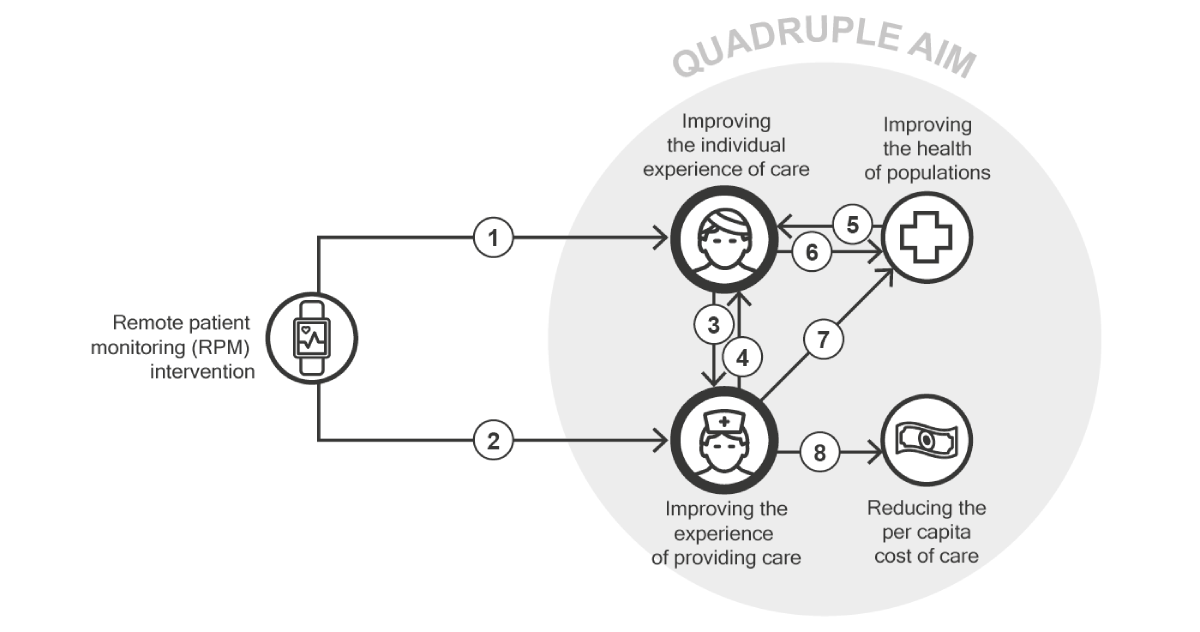
Schematic overview of the relations between patient and staff experience in RPM and the other components of the Quadruple Aim framework. Each arrow symbolizes a relation.

Many characterizations of patient and staff experience exist [17-19], some of which distinguish between determinants of experience and experience manifestations [20]. For our review, we maintained this distinction, as we aimed to focus on the broad spectrum of factors affecting and affected by patient and staff experience. To do so, we adopted the general conceptualization of patient and staff experience as characterized in the Quadruple Aim, a widely used framework for health system optimization centered around 4 overarching goals: improving the individual experience of care, improving the experience of providing care, improving the health of populations, and reducing the per capita cost of care [21]. Adopting a Quadruple Aim perspective allows health system researchers and innovators to recognize not only the importance of patient and staff experience in their own rights but also the inextricable relations of these 2 goals to the other dimensions of health system performance [22]. To clarify the nature of these relations in the RPM domain, we provide a schematic overview in Figure 1.

Next, we refer to the numbers in Figure 1 to touch upon prominent relationships between patient and staff experience in RPM within the Quadruple Aim framework and provide examples of experience constructs relevant to each relationship:

- Numbers 1 and 2: The characteristics of specific RPM interventions directly affect the patient and staff experience. Examples of experience constructs related to this mechanism are expressed in terms of *usability* or *wearability*, which are attributes of systems or products contributing to the care experience of patients and the work experience of staff.
- Numbers 3 and 4: Patient and staff experiences relate to each other through care delivery. Human connections, especially in the form of carer-patient relationships, represent a major factor in both patient and staff experience. An example of experience constructs related to this mechanism is expressed in terms of the *quality of the relationship*.
- Numbers 5 and 6: A major determinant of patient experience is represented by the health outcomes achieved as a result of the received care. An example of a measure of quality related to this mechanism is expressed in terms of the *quality of life*, which is an attribute of patient experience directly affected by a patient’s health status. In contrast, patient experience itself is a determinant of the clinical effectiveness of RPM interventions. For example, the patient experience afforded by a given intervention is a determinant of both *adoption* of and *adherence* to that intervention, ultimately affecting its clinical impact. In a recent review, for instance, low patient adherence was identified as the main factor associated with ineffective RPM services [23].
- Number 7: Similarly, staff experience can be a determinant of clinical effectiveness. Experience-related issues, such as *alarm fatigue*, contribute to medical errors and lower the quality of care delivery [24].
- Number 8: Staff experience can also impact the cost of care. For example, the *time effort* required for the use of a given intervention can constitute a source of extra costs. More indirectly, low staff *satisfaction* and excessive *workload* can increase health care staff turnover, resulting in additional expenses at the level of the health system.

Overall, the overview in Figure 1 can help us grasp the nuances of the role of patient and staff experience on the overall impact of RPM interventions, as well as the importance of measuring experience factors, not only in isolation, but also in relation to other dimensions of care quality. In this review, we therefore covered a broad range of experience-relevant factors, including both experiential determinants (eg, usability) and manifestations (eg, adherence). Overall, this study aimed to obtain a comprehensive set of experience constructs and corresponding measurement instruments used in contemporary RPM research and to propose an initial set of guidelines for improving methodological standardization in this domain.

## Methods

### Protocol Registration and PRISMA Guidelines

The study protocol was registered in the PROSPERO (International Prospective Register of Systematic Reviews) database (CRD42021250707). This systematic review adhered to the PRISMA (Preferred Reporting Items for Systematic Reviews and Meta-Analyses) guidelines. The PRISMA checklist is provided in Multimedia Appendix 1 [25].

### Criteria for Study Eligibility

Our study population consisted of adult (≥18 years old) patients and staff members involved as participants in reported RPM evaluations. Full-text papers reporting instances of patient and staff experience measuring in RPM interventions, written in English, and published after January 1, 2011, were considered for eligibility.

For the scope of our review, we considered as RPM any intervention possessing the following characteristics:

- Sensor-based patient monitoring, intended as the use of at least 1 sensor to collect patient information at a distance. Therefore, we excluded interventions that were purely based on the collection of “sensor-less” self-reported measures from patients. This is because we believe the use of sensors constitutes a key element of RPM and one that strongly contributes to experiential aspects in this domain. However, we adopted a broad definition of “sensor,” considering as such, for instance, smartphone cameras (eg, postoperative wound-monitoring apps) and analog scales or thermometers (eg, interventions relying on patients submitting manually entered weights or temperatures). By “at a distance,” we meant not only cases in which data were transferred from nonclinical environments, such as home monitoring, but also cases such as tele–intensive care units (tele-ICUs), in which data were transferred from one clinical environment to another. Furthermore, we included interventions relying on both continuous and intermittent monitoring.
- Clinical decision-making as an intended use of remotely collected data. Therefore, we excluded interventions in which the collected data were meant to be used exclusively for research purposes and not as a stage of development of an RPM intervention to be adopted in patient care. For instance, we excluded cases in which the remotely collected patient data were only used to test research hypotheses unrelated to the objective of implementing RPM interventions (eg, for drug development purposes). This is because in this review we were interested in RPM as a tool for the provision of remote patient care, rather than as an instrument for research. We also excluded interventions in which patients themselves were the only recipients of the collected data and no health care professional was involved in the data analysis and use.

Furthermore, we excluded:

- Evaluations of attitudes, not interventions: contributions in which only general attitudes toward RPM in abstract were investigated, rather than 1 or more specific RPM interventions.
- Not reporting any evaluation: contributions not focusing on the evaluation of 1 or more specific RPM interventions, for instance, papers providing theoretical perspectives on the field (eg, research frameworks or theoretical models).
- Evaluation of technology, not interventions: contributions only focused on evaluating RPM-related technology, for instance, papers focused on testing sensors, software, or other service components in isolation rather than as a part of any specific RPM intervention.
- Not just RPM: contributions not specifically focused on RPM but including RPM interventions in their scope of research, for instance, papers reporting on surveys obtained from broad cohorts of patients (including RPM recipients) in a noncontrolled way. An example of such contributions would be represented by studies focusing on patient experience with mobile health apps in general, covering both interventions involving RPM and interventions not including any kind of patient monitoring, without a clear way to distinguish between the 2 kinds of interventions in the contribution results. This was chosen in order to maintain the review focus on RPM interventions. Instead, papers including both RPM and other forms of care provisions within the same intervention were included, as well as papers comparing RPM to non-RPM interventions in a controlled way.
- Primary care intervention only: interventions only involving general practitioners (GPs) and other primary care practitioners as health care providers of the RPM intervention. This is because we expected marked differences between the implementation of RPM in primary care and at other levels of care, due to deep dissimilarities in settings, workflows, and routines. Examples of RPM interventions *only* involving primary care providers included kiosk systems (for which a common measuring point was provided to many patients) or pharmacy-managed medication-monitoring programs. RPM interventions involving primary care providers *and* providers from higher levels of care, however, were included in the review.
- Staff-to-staff intervention: contributions reporting on interventions exclusively directed at staff, for instance, papers reporting on RPM methods aimed at monitoring stress levels of health care workers.
- Target group other than patient or staff: contributions aimed at collecting experience measures in target groups other than patients or staff, for instance, papers investigating the experience in RPM for informal caregivers.

### Search Method

To identify relevant publications, the following electronic databases were searched: (1) Medline (PubMed) and (2) EMBASE. Search terms included controlled terms from Medical Subject Headings (MeSH) in PubMed and Emtree in EMBASE, as well as free-text terms. Query term selection and structuring were performed collaboratively by authors VP, HCMO, and PG (who is a clinical librarian at the Leiden University medical library). The full search strategies are reported in Multimedia Appendix 2. Because the aim of the review was to paint a contemporary picture of experience measures used in RPM, only studies published starting from January 1, 2011, were included.

### Study Selection

**Figure 2:**
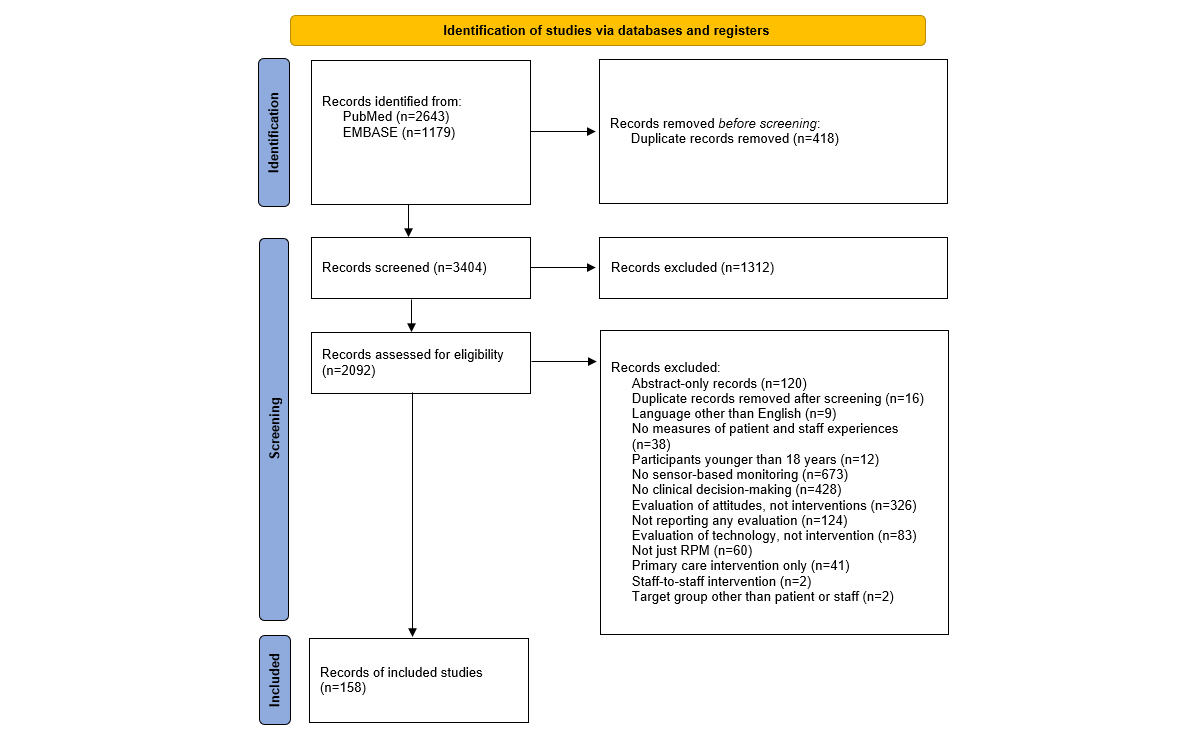
Flowchart of study selection. RPM: remote patient monitoring.

Study selection was performed by VP and HCMO, who used Rayyan, an online research tool for managing review studies [26], to independently screen both titles and abstracts in the initial screening and full texts in the final screening. Discrepancies were solved by discussion. A flowchart of study selection is depicted in Figure 2.

### Quality Appraisal

The objective of this review was to provide a comprehensive overview of the relevant literature, rather than a synthesis of specific intervention outcomes. Therefore, no papers were excluded based on the quality appraisal, in alignment with similar studies [27].

### Data Extraction and Management

Data extraction was performed independently by VP and HCMO. The extraction was performed in a predefined Microsoft Excel sheet designed by VP and HCMO. The sheet was first piloted in 15 included studies and iterated upon to optimize the data extraction process. The full text of all included studies was retrieved and uploaded in the Rayyan environment. Next, the full text of each included study was examined and relevant data were manually inputted in the predefined Excel sheet. Discrepancies were resolved by discussion. The following data types were extracted: (1) general study information (authors, title, year of publication, type of study, country or countries); (2) target disease(s), intervention, or clinical specialty; (3) used patient or staff experience evaluation instrument and measured experience construct; (4) evidence base, if indicated; and (5) number of involved staff or patient participants. By “construct,” we referred to the “abstract idea, underlying theme, or subject matter that one wishes to measure using survey questions” [28]. To identify the measured experience construct, we used the definition provided in the source contribution, whenever available.

### Data Analysis

First, we analyzed the collected data through building general overviews depicting the kind of target participants (patients or staff) of the collected experience measures and their use over time. To organize the diverse set of results collected through the systematic review, we then performed a correspondence analysis (CA) [29], a multivariate statistical technique used for exploring and displaying relationships between categorical data. CA transforms a 2-way table of frequencies between a row and a column variable into a visual representation of relatedness between the variables. This relatedness is expressed in terms of inertia, which represents “a measure of deviation from independence” [30] between the row and column variables. Any deviations from the frequencies expected if the row and column variables were completely independent from each other contribute to the total inertia of the model. CA breaks down the inertia of the model by identifying mutually independent (orthogonal) dimensions on which the model inertia can be represented. Each successive dimension explains less and less of the total inertia of the model. On each dimension, relatedness is expressed in terms of the relative closeness of rows to each other, as well as the relative closeness of columns to each other. CA has been previously used to find patterns in systematic review data in the health care domain [31].

In our case, a 2-way table of frequencies was built based on how often any given instrument (eg, System Usability Scale [SUS]) was used to measure any given construct (eg, usability) in the included literature. Therefore, in our case, the total inertia of the model represented the amassed evidence base for relatedness between the collected experience constructs and measures, based on how they were used in the included literature.

To build the table of frequencies, the data extracted from the systematic review underwent a round of cleaning, in which the formulation of similar constructs was made more homogeneous: for instance, “time to review,” “time to response,” and “time for task” were merged under 1 label, “time effort.” An overview of the merged construct formulations is provided in Multimedia Appendix 3. The result of the CA was a model where 2 dimensions contributed to more than 80% of the model’s inertia (explaining 44.8% and 35.7%, respectively) and where none of the remaining 59 dimensions contributed more than 7.3% to the remaining inertia. This gap suggests the first 2 dimensions to express meaningful relationships that are not purely based on random variation. A 2D solution was thus chosen.

## Results

### General Observations

A total of 158 studies reporting at least 1 instance of patient or staff experience measuring in RPM were included in the review. The included studies covered a broad range of RPM interventions, most prominently diabetes care (n=30, 19%), implanted devices (n=12, 7.6%), and chronic obstructive pulmonary disease (COPD; n=10, 6.3%). From these studies, we reported 546 experience-measuring instances in RPM, covering 160 unique experience-measuring instruments used to measure 120 unique experience constructs.

Our results included 4 kinds of versatile (intended as nonspecific) experience-measuring instruments: the custom survey, log file analysis, protocol database analysis, and task analysis. All of them can be used for measuring disparate kinds of constructs:

- By “custom survey,” we refer to survey instruments created to evaluate patient or staff experience in connection to 1 specific RPM study and only for that study.
- By “log file analysis,” we refer to the set of experience assessment methods based on the automatic collection of data through the RPM digital infrastructures themselves [32]; examples are clicks, uploads, views, or other forms of interactions between users and the RPM digital system. This set of methods is typically used to estimate experience-relevant constructs, such as adherence and compliance.
- By “protocol database analysis,” we refer to the set of experience assessment methods based on the manual collection of data performed by RPM researchers within a specific research protocol; an example of a construct measured with these instruments is the willingness to enroll.
- By “task analysis,” we refer to the set of experience assessment methods based on the real-life observation of users interacting with the RPM system [33].

In addition to these 4 instruments, our results included a large number of specific instruments, such as standard indexes, surveys, and questionnaires. Overall, the most frequently reported instrument was, by far, the custom survey (reported in 155/546, 28.39%, instances), while the most frequently reported experience construct was satisfaction (85/546, 15.57%), closely followed by quality of life (71/546, 13%).

### Target Participants and Timeline

**Figure 3:**
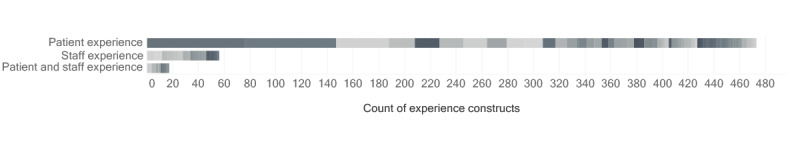
Count of mentioned instances of experience constructs organized by target participant: patient, staff, or both. Different shades of gray indicate different constructs.

We found large differences in the number of RPM-relevant experience constructs and instruments used for patients and for staff (see Figure 3). We also found instruments used for both patients and staff. Either these were broadly used instruments (eg, the SUS) that were administered to both patients and staff within the same study, or they were measures of interactions between patients and staff (eg, log file analysis instruments recording the number of remote contacts between patients and nursing assistants).

RPM research appears to focus much more on patient experience than on staff experience, which was investigated in only 20 (12.7%) of the 158 included papers. Although it is possible that our exclusion criteria contributed to the paucity of staff experience measures, only 2 (0.1%) of 2092 studies were excluded for reporting on interventions directed exclusively at staff. Of the 41 (2%) studies we excluded for reporting on primary care interventions, we found 6 (15%) studies reporting on staff experience, a rate comparable to the one in the included sample. Furthermore, although our choice to exclude papers reporting on the RPM experience of informal caregivers might have contributed to a reduction in the number of collected constructs and measures, only 2 (0.1%) of 2092 studies were excluded for this reason, and the constructs used in these contributions were not dissimilar from the ones found in the included literature.

Among the included contributions that did investigate staff experience, we noticed that the number of participant staff members involved in the reported studies was only reported in a minority of cases (9/20, 45%).

**Figure 4:**
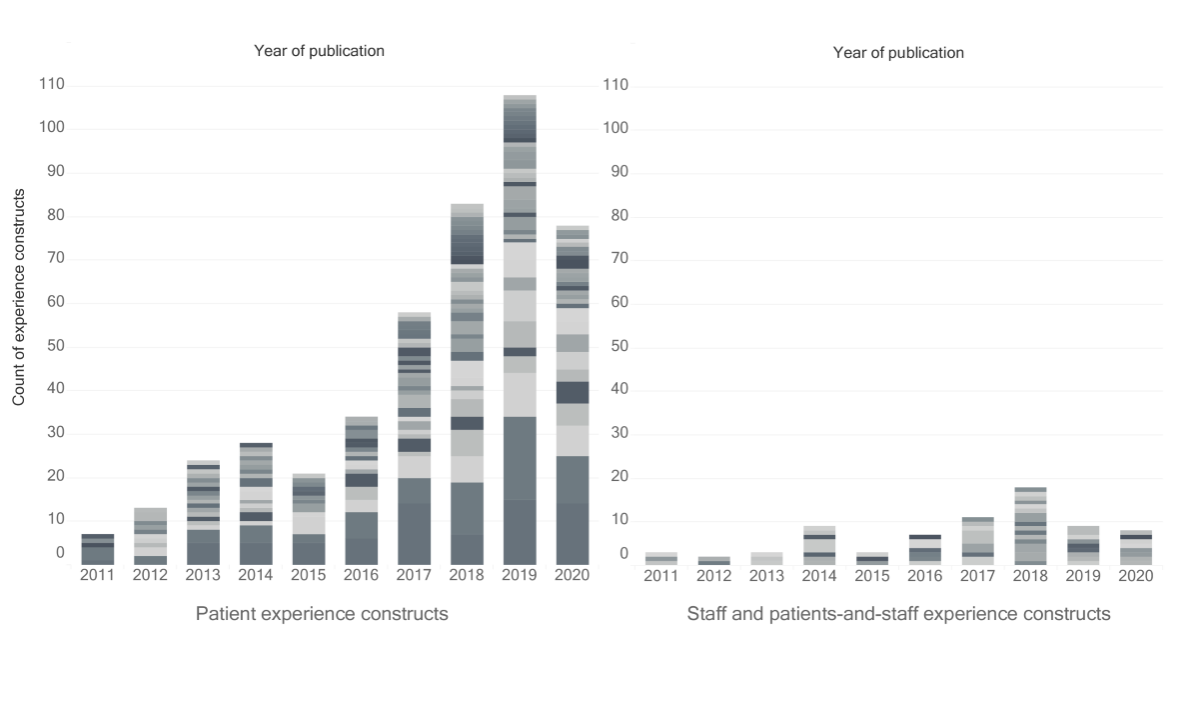
Count of mentioned instances of experience constructs for patients (left) or for staff, and patients and staff (right) in the included literature from 2011 to 2020. Different shades of gray indicate different constructs.

Furthermore, a time-based overview of the collected results (Figure 4) provided us with an impression of the expansion of the field in the time frame of interest for both patient and staff experience measures.

### Correspondence Analysis

**Figure 5:**
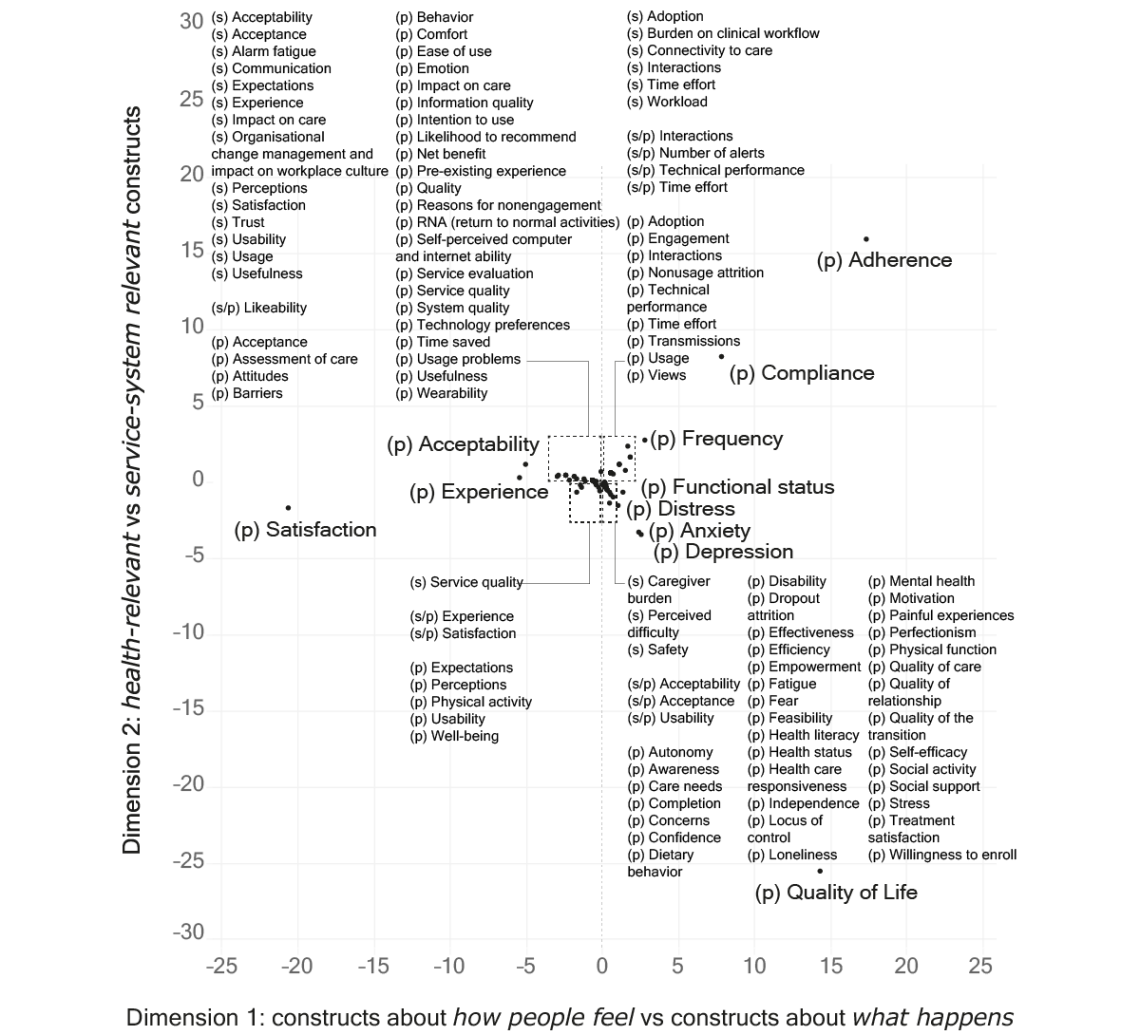
Graphical display of the results of the CA, visualized on the coordinates of dimensions 1 and 2. The labels (s), (p), and (s/p) refer to experience constructs used for staff, patients, and both. CA: correspondence analysis.

The plotted results of the CA of experience constructs are shown in Figure 5. Here, we discuss the outlook and interpretation of each dimension.

The first dimension explained more than 44% of the model’s inertia. The contributions of this dimension showed which constructs had the most impact in determining its orientation: satisfaction (36%) and to a lesser extent adherence (26%) and quality of life (17%). On the negative (left) side of this dimension, we found constructs such as satisfaction, perceptions, and acceptability, which are associated with subjective measures of patient and staff experience and relate to how people feel or think in relation to RPM interventions. On the positive (right) side of this dimension, we found constructs such as adherence, compliance, and quality of life, which are associated with objectivized measures of patient and staff experience. By “objectivized measures,” we referred to measures that are meant to capture phenomena in a factual manner, ideally independently from personal biases and subjective opinions. Adherence and compliance, particularly, are often measured through passive collection of system data (eg, log file analysis) that reflect objective measures of the way patients or staff interact with RPM propositions. Even in the case of (health-related) quality of life, which can include subjective connotations and components, measures usually aim at capturing an estimation of the factual impact of health status on a person’s overall life quality.

In this sense, we attributed a distinction between *how people feel* versus *what happens* experience constructs to this first dimension. We noted that a similar distinction (between subjective vs objective measures of engagement in remote measurement studies) was previously proposed as a meaningful differentiation to structure “a field impeded by incoherent measures” [27].

The second dimension explained 35% of the model’s inertia. The contributions of this dimension showed which constructs had the most impact in determining its orientation: quality of life (62%) and adherence (24%). On the negative (bottom) side of this dimension, we found constructs such as quality of life, depression, and anxiety, which are often used as experiential descriptors of health outcomes. On the positive (top) side of this dimension, we found adherence, compliance, and frequency, which are often used as descriptions of the interactions of patients or staff with a specific (RPM) system. Thus, we attributed a distinction between *health-relevant* versus *system-relevant* experience constructs to this second dimension.

**Figure 6:**
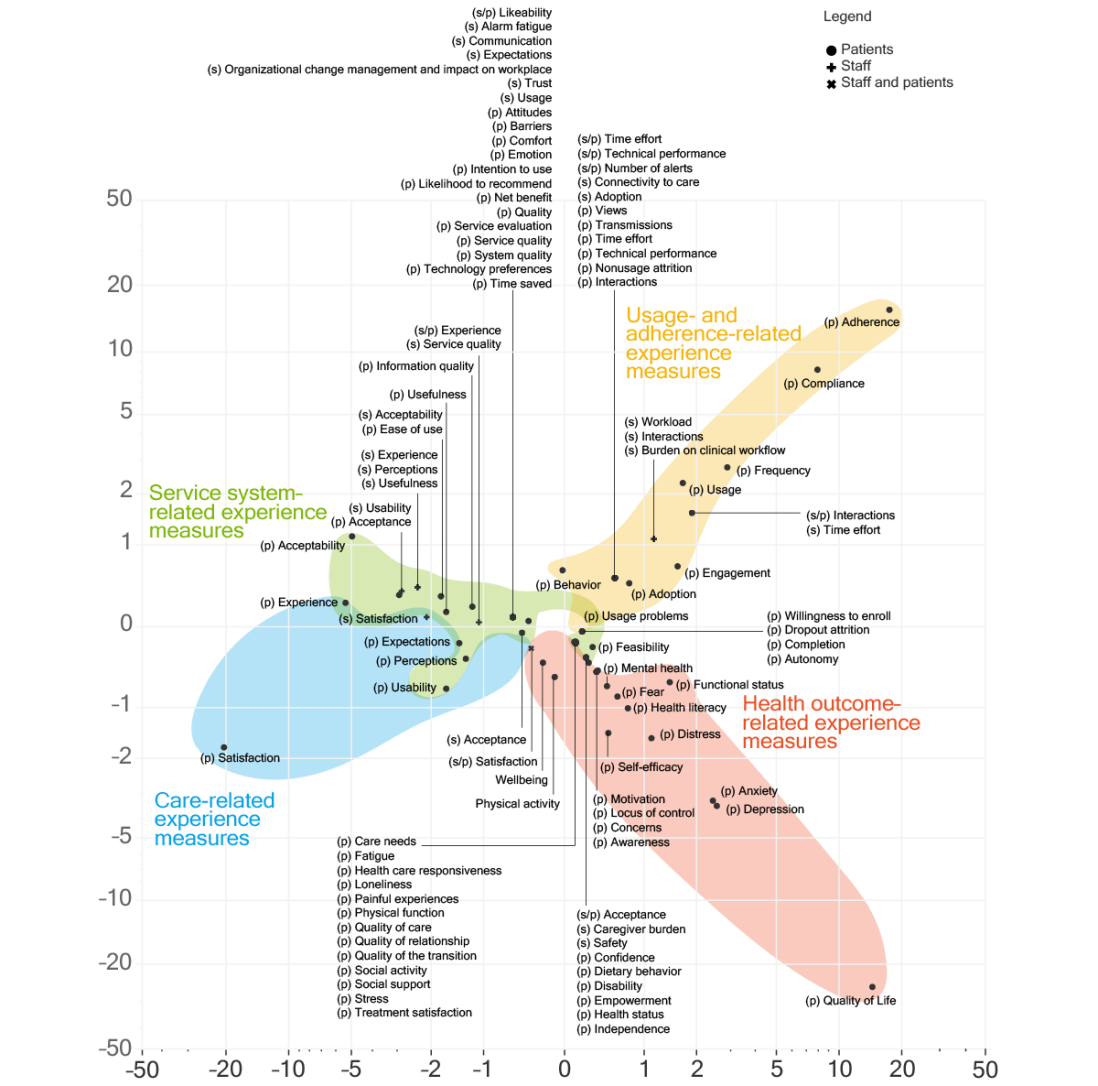
Clustered results of the CA (displayed on a logarithmic scale). CA: correspondence analysis.

Based on the results of CA, we proposed a categorization of patient and staff experience–related constructs into 4 partly overlapping clusters. Coherent with the offered explanation of the 2 dimensions and in consideration of the constructs found in each area, we labeled these as service system–related experience measures, care-related experience measures, usage- and adherence-related experience measures, and health outcome–related experience measures. In Figure 6, we display the results of the CA labeled through this categorization. In this second visualization, we presented the results on a logarithmic scale to improve the visibility of constructs close to the center of the axes. Overall, this categorization of patient and staff experience constructs used in the RPM literature paints a landscape of the contemporary research in this field, which shows a mix of influences from clinical disciplines, health psychology, human factors engineering, service design, user research, systems engineering, and computer science.

**Figure 7:**
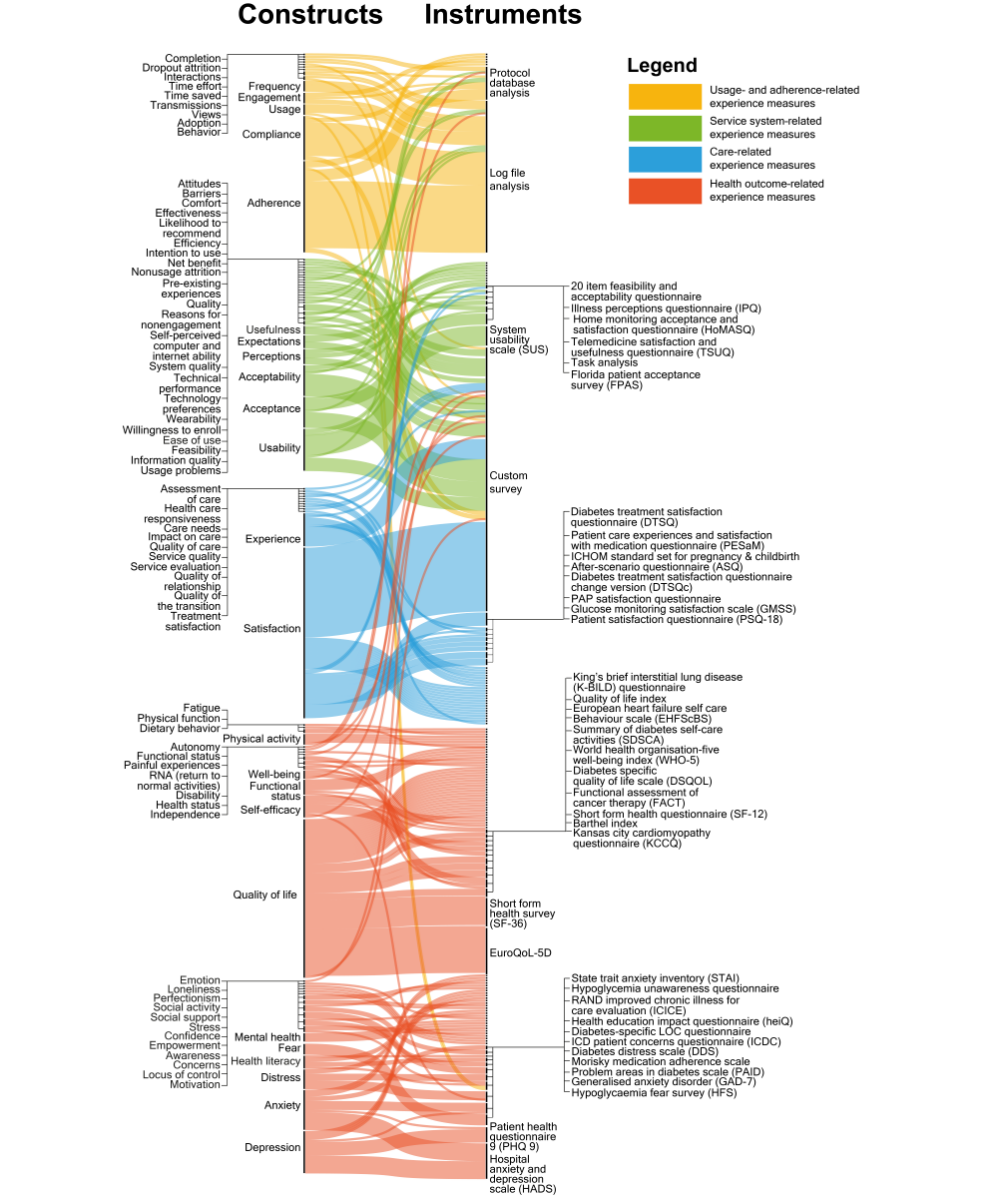
Reported patient experience constructs (left) and some associated measuring instruments (right). The thickness of each line refers to the number of instances each construct was used in the included studies. A complete version of this visual can be found in Multimedia Appendix 4, and an interactive version can be found in Ref [34].

A visualization of the reported patient experience constructs and some of the related measuring instruments, organized by the categories identified in the CA, is available in Figure 7. A complete version of this visual can be found in Multimedia Appendix 4, and an interactive version can be found in [34]. In this figure, we can note the limited crossovers between constructs belonging to different categories, with the exception of versatile instruments, such as custom survey and log file analysis.

### Recommendations

**Figure 8:**
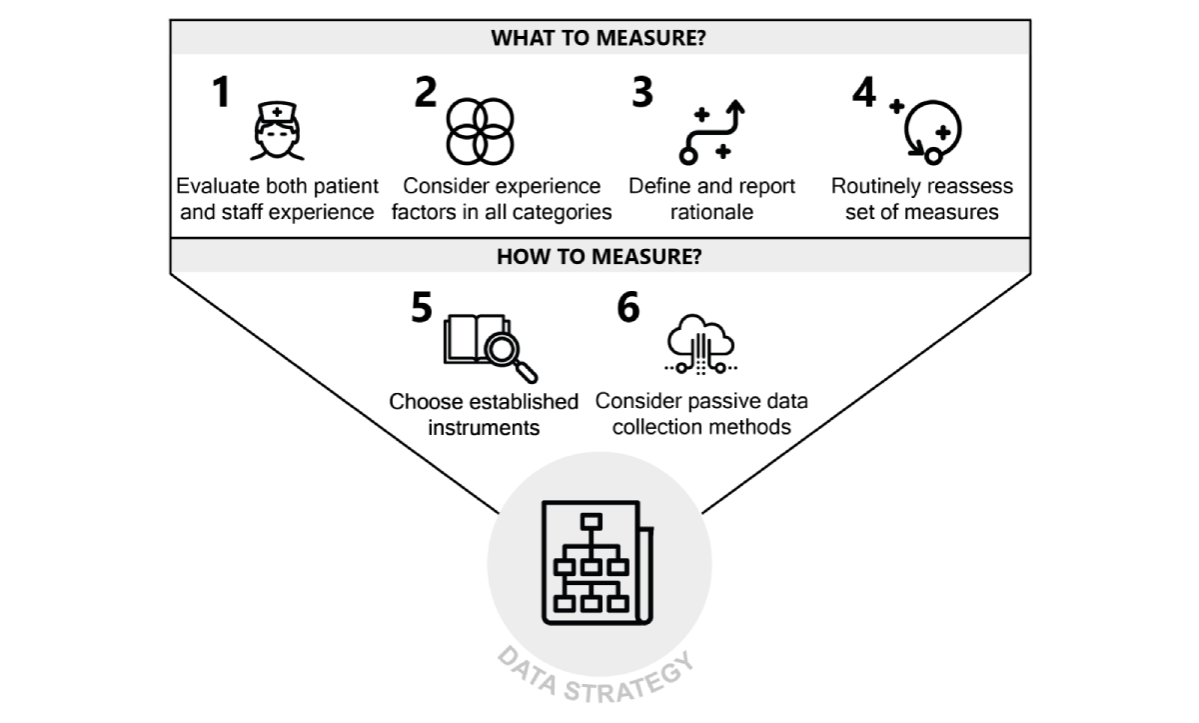
Recommendations for patient and staff experience measuring in the RPM domain. RPM: remote patient monitoring.

In the light of the collected findings, here we provide a set of recommendations to RPM patient and staff experience evaluators, in terms of both what to measure and how to measure it (Figure 8). Although these recommendations are functional to strengthen the quality of individual research protocols, they are also meant to stimulate increased standardization in the field as a whole.

Regarding what to measure, we provide 4 main recommendations. The first is to conduct structured evaluations of staff experience next to patient experience. Failing to evaluate staff experience leads to risks, such as undetected staff nonadherence, misuse, and overworking. Although new competencies need to be developed in order for staff to unlock the untapped potential of RPM [35], seamless integration with existing clinical workflows should always be pursued and monitored.

The second recommendation is to consider experience constructs in all 4 clusters indicated in Figure 6, as these represent complementary facets of an overall experiential ensemble. Failing to do so exposes RPM evaluators to the risk of obtaining partial information (eg, only shedding light on *how people feel* but not on *what happens* in terms of patient and staff experience in RPM).

The third recommendation is to explicitly define and report a clear rationale regarding which aspects of patient and staff experience to prioritize in evaluations, depending on the goals and specificities of the RPM intervention. This rationale should ideally be informed by preliminary qualitative research and by a collaborative mapping of the expected relationships between patient and staff experience and other components of the Quadruple Aim framework for the RPM intervention at hand. Failing to follow this recommendation exposes RPM evaluators to the risk of obtaining results that are logically detached from each other and as such cannot inform organic improvement efforts. Virtuous examples of reporting a clear rationale were provided by Alonso-Solís et al [36] and den Bakker et al [37], who offered detailed accounts of the considerations used to guide the selection of included experience measures. Several existing frameworks and methods can be used to map such considerations, including the nonadoption, abandonment, scale-up, spread, and sustainability (NASSS) framework [38] and the logical framework [39]. A relatively lightweight method to achieve such an overview can also be represented by the use of Figure 1 as a checklist to inventory possible Quadruple Aim relationships for a specific RPM intervention.

The fourth recommendation is to routinely reassess the chosen set of experience measures after each iteration of the RPM intervention design. Initial assumptions regarding relationships between experience factors and other dimensions of intervention quality should be verified once the relevant data are available, and new ones should be formulated, if necessary. If the RPM intervention transitions from research stages to implementation as the standard of care, it is recommended to keep on collecting at least some basic experience measures for system quality monitoring and continuous improvement. Failing to update the set of collected measures as the RPM intervention progresses through successive development stages exposes RPM evaluators to the risk of collecting outdated information, hindering iterative improvement processes.

Regarding how to measure RPM patient and staff experience, we provide 2 main recommendations. The first is to work with existing, validated and widely used instruments as much as possible, only creating new instruments after a convincing critique against current ones. Figure 7 can be used to find existing instruments measuring a broad range of experience-relevant constructs so as to reduce the need to create new ones.

For instance, researchers interested in evaluating certain experience constructs, ideally informed by preliminary qualitative research, might consult the full version of Figure 7 (available in Multimedia Appendix 4 or as an interactive map in Ref. [34]) to find their construct of interest on the left side of the graph, follow the connecting lines to the existing relevant measures on the right, and identify the most frequently used ones. They can also use the visual to consider other possibly relevant constructs.

Alternatively, researchers can use the open access database of this review [40] and especially the “extracted data” Excel file to search for the construct of interest and find details of papers in the RPM domain in which the construct was previously measured.

Failing to follow this recommendation exposes RPM researchers to the risk of obtaining results that cannot be compared to meaningful benchmarks, compared to other RPM interventions, or be included in meta-analyses.

The second recommendation is to consider adopting automatic, “passive” methods of experience data collection, such as the ones we referred to in this review as log file analysis, so as to obtain actionable estimates of user behavior with a reduced need for patients and staff to fill tedious surveys [41] or otherwise provide active input. Failing to consider automatically collected log file data on patient and staff experience constitutes a missed opportunity in terms of both the quality and cost of evaluation data. We recognize such nascent data innovations as promising [42] but also in need of methodological definition, particularly in terms of an ethical evaluation of data privacy and access [43,44] in order to avoid exploitative forms of prosumption [45].

## Discussion

### Principal Findings

This study resulted in a structured overview of patient and staff experience measures used in contemporary RPM research. Through this effort, we found that the research landscape has seen a sizeable growth in the past 10 years, that it is affected by a relative lack of focus on staff experience, and that the overall corpus of collected measures can be organized in 4 main categories (service system–related, care-related, usage- and adherence-related, and health outcome–related experience measures). Little to no consensus or standardization was found in the adopted methods. Based on these findings, a set of 6 actionable recommendations for RPM experience evaluators was provided, with the aim of improving the quality and standardization of experience-related RPM research. The results of this review align with and expand on recent contributions in the field, with particular regard to the work of White et al [27].

### Directions for Further Research

Fruitful future research opportunities have been recognized in various areas of RPM experience measuring. Among them, we stress the need for comparative studies investigating patient and staff experience factors across different RPM interventions; for studies clarifying the use, potential, and limitations of log file analysis in this domain; and (most importantly) for studies examining the complex relationships between experience factors, health outcomes, and cost-effectiveness in RPM.

Ultimately, we recognize the need for integrated data strategies for RPM, intended as processes and rules that define how to manage, analyze, and act upon RPM data, including continuously collected experience data, as well as clinical, technical, and administrative data. Data strategies can represent a way to operationalize a systems approach to health care innovation, described by Komashie et al [46] as “a way of addressing health delivery challenges that recognizes the multiplicity of elements interacting to impact an outcome of interest and implements processes or tools in a holistic way.” As complex, adaptive, and partly automated systems, RPM interventions require sophisticated data strategies in order to function and improve [47]; continuous loops of system feedback need to be established and analyzed in order to monitor the impact of RPM systems and optimize their performance over time, while respecting patients’ and staff’s privacy. This is especially true in the case of RPM systems including artificial intelligence (AI) components, which require continuous monitoring and updating of algorithms [48-50]. We characterize the development of integrated, interdisciplinary data strategies as a paramount challenge in contemporary RPM research, which will require closer collaboration between digital health designers and health care professionals [51-53]. We hope to have provided a small contribution to this overall goal through our effort to structure the current landscape of RPM patient and staff experience evaluation.

### Strengths and Limitations

We acknowledge both strengths and limitations of the chosen methodologies. The main strength of this review is its extensive focus, covering a large number of experience measures and RPM interventions. However, a limitation introduced by such a broad scope is the lack of differentiation by targeted condition, clinical specialty, RPM intervention characteristics, geographical area, or other relevant distinctions. Furthermore, limitations were introduced by choices, such as focusing exclusively on contributions in English and on nonprimary care and nonpediatric RPM interventions.

### Conclusion

Contemporary patient and staff experience measuring in RPM is affected by a lack of consensus and standardization, affecting the quality of both primary and secondary research in this domain. This issue determines a critical knowledge gap in our understanding of the effectiveness of RPM interventions, which are known to bring about radical changes to the care experience of both patients and staff. Bridging this knowledge gap appears to be critical in a global context of urgent need for increased resource effectiveness across health care systems, including through the increased adoption of safe and effective RPM. In this context, this review offers support for RPM experience evaluators by providing a structured overview of contemporary patient and staff experience measures and a set of practical guidelines for improving research quality and standardization in this domain.

## Appendix

Multimedia Appendix 1 PRISMA (Preferred Reporting Items for Systematic Reviews and Meta-Analyses) checklist.

Multimedia Appendix 2 Full search strategies.

Multimedia Appendix 3 Overview of the merged construct formulations .

Multimedia Appendix 4 Reported patient experience constructs and associated measuring instruments (complete visual).

## Abbreviations

CA: correspondence analysis
PRISMA: Preferred Reporting Items for Systematic Reviews and Meta-Analyses RPM: remote patient monitoring
SUS: System Usability Scale

## Data Availability

The data sets generated and analyzed during this review are available as open access in Ref. [40].
