# Supplementary figures and images for: Patient and Staff Experience of Remote Patient Monitoring—What to Measure and How: Systematic Review
Source: J Med Internet Res. 2024 Apr 22;26:e48463. doi: 10.2196/48463 (PMC11074906; doi:10.2196/48463)

## Multimedia Appendix 1


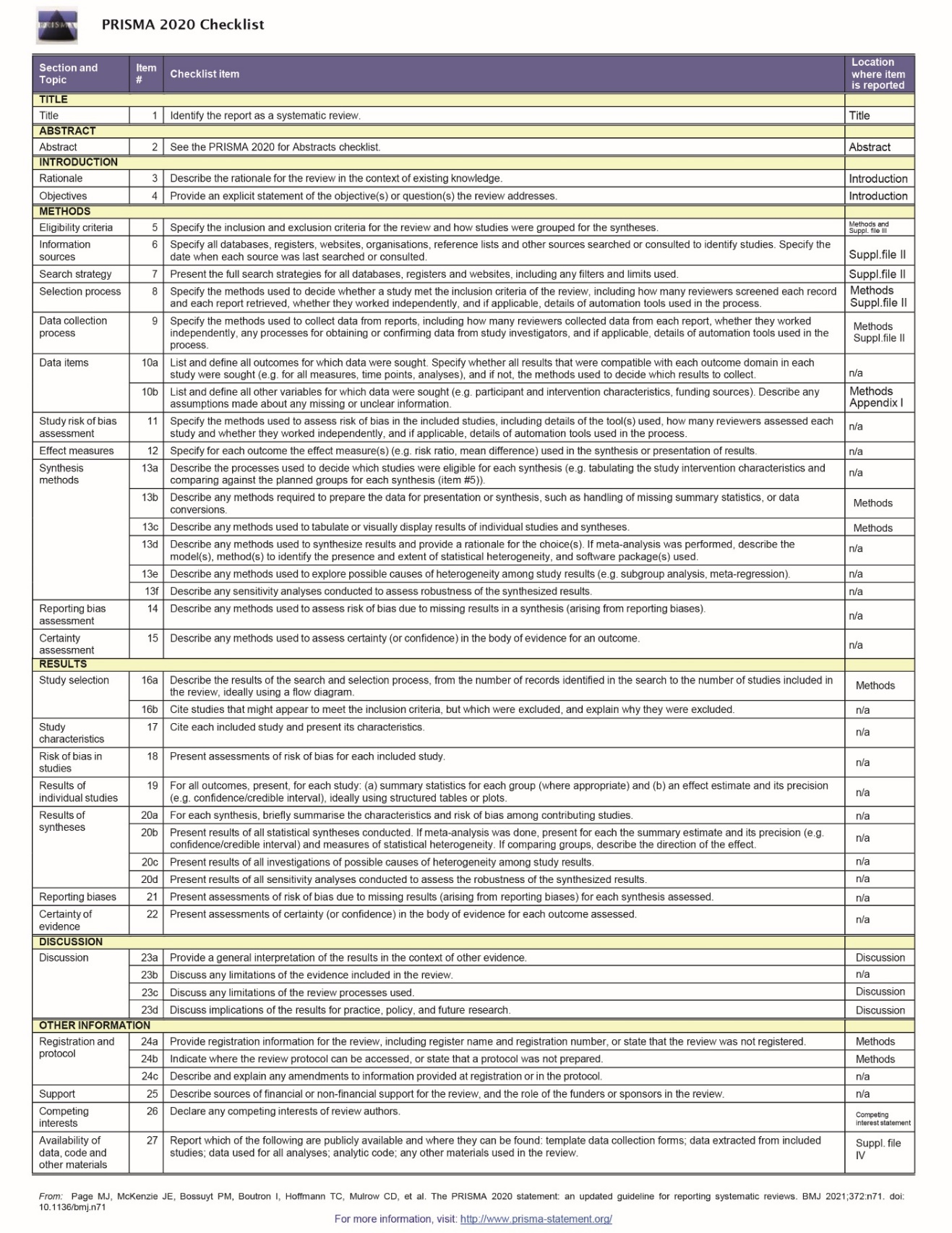

Supplement: Multimedia Appendix 1 [file jmir_v26i1e48463_app1.docx]
