# Supplementary material for: Patient and Staff Experience of Remote Patient Monitoring—What to Measure and How: Systematic Review
Source: J Med Internet Res. 2024 Apr 22;26:e48463. doi: 10.2196/48463 (PMC11074906; doi:10.2196/48463)
Supplement: Multimedia Appendix 2 [file jmir_v26i1e48463_app2.docx]

## Multimedia Appendix 2

For both databases, the sets of keywords for each concepts were first searched separately.

Concept 1: Remote Patient Monitoring

Concept 2: Measures and indicators

Concept 3: Staff experience

Concept 4: Patient experience

Following, the separate searches were merged through Boolean operators following the scheme:

*[Concept 1] AND [Concept 2] AND (Concept 3 OR Concept 4)*

to find all papers containing mentions of patient experience measures used in RPM research, staff experience measures used in RPM research, or both.

The search entries for both databases are reported hereafter. The query was performed on both databases on the 12th of February 2021, including papers published from the 1st of January 2011.

### 1.2 Medline (PubMed) search entry

CONCEPT 1. Remote Patient Monitoring

"telemedicine"[majr] OR "telemedicine*"[tiab] OR "telehealth"[tiab] OR "Wearable Electronic Devices" [majr] OR “Wearable Electronic Device*” [tiab] OR “Wearable Electronic*”[tiab] OR “Wearable Technolog*”[tiab] OR “Wearable Device*”[tiab] OR “Wearable computer*”[tiab] OR "Wireless Technology"[majr] OR “Wireless Technolog*”[tiab] OR “Wireless sens*” [tiab] OR “Wireless monitor*” [tiab] OR "Monitoring, Physiologic"[majr] OR “physiologic monitor*”[tiab] OR “vital signs monitoring*”[tiab] OR “Patient Monitor*”[tiab] OR “self-monitor*”[tiab] OR “self monitor*”[tiab] OR “continuous wireless monitor*”[tiab] OR “continuous monitor*” [tiab] OR “remote monitor*”[tiab] OR "Remote Sensing Technology"[majr] OR “Remote Sensing*”[tiab]

CONCEPT 2. Measures and indicators

"Quality Indicators, Health Care"[majr] OR "Outcome and Process Assessment, Health Care"[majr] OR "Patient Reported Outcome Measures"[Majr] OR “Patient Reported Outcome Measure*”[tiab] OR proms [tiab] OR “Patient reported Outcome*”[tiab] OR “Patient-reported Outcome*”[tiab] OR “Patients reported Outcome*” [tiab] OR “Patients-reported Outcome*” [tiab] OR “patient-reported experience measure*” [tiab] OR “patient reported experience measure*” [tiab] OR "Surveys and Questionnaires" [Majr] OR “survey*” [tiab] OR “measurement instrument*”[tiab] OR “assessment tool*” [tiab] OR “measurement tool*” [tiab] OR “assessment instrument*” [tiab] OR “variable*” [tiab]

CONCEPT 3. Staff experience

ergonomics [majr] OR Ergonomic* [tiab] OR “Cognitive Ergonomic*”[tiab] OR “Engineering Psychology” [tiab] OR "Attitude of Health Personnel"[majr] OR "Attitude to Computers"[majr] OR “attitude of health personnel” [tiab] OR “staff attitude*” [tiab] OR “staff acceptance*” [tiab] OR "Alert Fatigue, Health Personnel"[majr] OR "alarm fatigue*"[tiab] OR "alert fatigue*"[tiab] OR "Burnout, Professional"[majr] OR "burnout*"[tiab] OR "Occupational Diseases"[majr] OR "occupational disease*"[tiab] OR "occupational risk*"[tiab] OR "occupational hazard*"[tiab] OR "occupational dysfunction*"[tiab] OR "Job Satisfaction"[majr] OR "job satisfaction*"[tiab] OR "professional satisfaction*"[tiab] OR (("Attitude" [majr] OR attitude* [tiab] OR acceptance* [tiab] OR "Perception"[majr] OR perception* [tiab] OR “staff experience*” [tiab] OR “physician experience*”[tiab] OR “physicians experience*”[tiab] OR “doctor experience*” [tiab] OR “doctors experience*” OR “nursing experience*” [tiab] OR “nurse experience*”[tiab] OR “nurses experience*” [tiab] OR “caregiver experience*” [tiab] OR “caregivers experience*” [tiab] OR "Personal Satisfaction" [majr] OR satisfaction [tiab] OR "Stress, Psychological"[majr] OR "Psychological Stress*"[tiab] OR "Occupational Stress"[Majr] OR "Occupational Stress*"[tiab]) AND ("Health Personnel"[majr] OR "personnel"[tiab] OR "Medical Staff, Hospital"[majr] OR staff*[tiab] OR "Nursing Staff, Hospital"[majr] OR nurs*[tiab] OR "Caregivers"[majr] OR caregiver*[tiab]))

CONCEPT 4. Patients experience

"Attitude to Health"[Majr] OR "Patient Satisfaction"[Majr] OR "Patient Satisfaction*" [tiab] OR “patient experience*” [tiab] OR patients experience* [tiab] OR "patient-centered care"[Majr] OR "patient-centered care" [tiab] OR "patient-centred care" [tiab] OR "Patient Acceptance of Health Care"[Majr] OR "Health Behavior"[Majr] OR ((ergonomics [majr] OR Ergonomic* [tiab] OR “Cognitive Ergonomic*”[tiab] OR “Engineering Psychology” [tiab] OR "Stress, Psychological"[majr] OR "Psychological Stress*"[tiab] OR "Attitude to Computers" [Majr] OR attitude* [tiab] OR acceptance* [tiab] or acceptabilit* [tiab] OR "Perception"[majr] OR “perception*”[tiab] OR "Emotions"[majr] OR “emotion*”[tiab] OR "Personal Satisfaction" [majr] OR satisfaction* [tiab] OR “usability” [tiab]) AND (patient* [tiab]))

### 1.2 EMBASE search entry

CONCEPT 1. Telemonitoring

*telehealth/ or *wearable computer/ or *wireless communication/ or *physiologic monitoring/ or *remote sensing/ or *telemedicine/ or (Wearable Electronic Device* or Wearable Electronic* or Wearable Technolog* or Wearable Device* or Wearable computer* or Wireless Technolog* or Wireless sens* or Wireless monitor* or physiologic monitor* or vital signs monitoring* or Patient Monitor* or self-monitor* or self monitor* or continuous wireless monitor* or continuous monitor* or remote monitor* or Remote Sensing* or telemedicine* or telehealth).ti,ab,kw.

CONCEPT 2. Measures and indicators

*health care quality/ or *patient-reported outcome/ or *outcome assessment/ or *health care survey/ or (Patient Reported Outcome Measure* or Proms or Patient reported Outcome* or Patient-reported Outcome* or Patients reported Outcome* or Patients-reported Outcome* or patient-reported experience measure* or patient reported experience measure* or measurement instrument* or assessment tool* or measurement tool* or assessment instrument* or variable*).ti,ab,kw.

CONCEPT 3. Staff experience

*attitude to computers/ or *"alert fatigue (health care)"/ or *professional burnout/ or *occupational disease/ or *job satisfaction/ or *mental stress or *job stress/ or

(Ergonomic* or Cognitive Ergonomic* or Engineering Psychology or attitude* of health personnel or staff attitude* or staff acceptance* or alarm fatigue* or alert fatigue* or burnout* or occupational disease* or occupational risk* or occupational hazard* or occupational dysfunction* or job satisfaction* or professional satisfaction* or attitude* or acceptance* or perception* or experience* or mental stress* or job stress*).ti,ab,kw or (*attitude/ or attitude*.ti,ab,kw. or *perception/ or perception*.ti,ab,kw. or *satisfaction/or satisfaction*.ti,ab,kw.) AND (*personnel/ or personnel.ti,ab,kw. or *staff/ or staff.ti,ab,kw. or *caregiver/ or caregiver*.ti,ab,kw.)

CONCEPT 4. Patient experience

*attitude to health/ or *health behavior/ or *patient satisfaction/ or (patient* satisfaction or patient experience* or patients experience* patient-centred care or patient cent?ed care).ti,ab,kw or ((*ergonomics/ or ergonomic*.ti,ab,kw or usability.ti,ab,kw or *attitude/ or *attitude to computers/ or attitude*.ti,ab,kw or *perception/ or perception*.ti,ab,kw or *mental stress/ or stress.ti,ab,kw or *emotion/ or emotion*.ti,ab,kw or *satisfaction/or satisfaction*.ti,ab,kw or acceptability.ti,ab,kw or acceptance*.ti,ab,kw) AND (patient*.ti,ab,kw))
