# Supplementary material for: Patient and Staff Experience of Remote Patient Monitoring—What to Measure and How: Systematic Review
Source: J Med Internet Res. 2024 Apr 22;26:e48463. doi: 10.2196/48463 (PMC11074906; doi:10.2196/48463)
Supplement: Multimedia Appendix 3 [file jmir_v26i1e48463_app3.docx]

| **Construct formulation as extracted** | **Construct formulation**  **after cleaning** |  |
| --- | --- | --- |
| User satisfaction | Satisfaction |  |
| Helpfullness | Usefulness |  |
| User-friendliness | Usability |  |
| Utility | Usefulness |  |
| User experience | Experience |  |
| Desired information | Information quality |  |
| Service evaluation | Service quality |  |
| Time to review | Time effort |  |
| Time to response | Time effort |  |
| Time for task | Time effort |  |
| Technical problems | Technical performance |  |
| Participation | Engagement |  |
| Proactive management | Engagement |  |
| Patient activation | Engagement |  |
| Usage and potential problems | Usage problems |  |
| Self-care agency | Self-efficacy |  |
| Self-care behavior | Self-efficacy |  |
| Health education | Health literacy |  |
| Knowledge | Health literacy |  |
| Functioning | Functional status |  |
| Functional recovery | Functional status |  |
| Mental status | Mental health |  |
| Use and adoption | Adoption |  |
| Uptake | Adoption |  |
